# Nostalgia and Heroism: Theoretical Convergence of Memory, Motivation, and Function

**DOI:** 10.3389/fpsyg.2020.577862

**Published:** 2020-12-23

**Authors:** Scott T. Allison, Jeffrey D. Green

**Affiliations:** ^1^ Department of Psychology, University of Richmond, Richmond, VA, United States; ^2^ Department of Psychology, Virginia Commonwealth University, Richmond, VA, United States

**Keywords:** nostalgia, heroism, heroic action, wisdom, motivation, inspiration, prosocial action

## Abstract

This article seeks to develop theoretical convergences between the science of nostalgia and the science of heroism. We take four approaches in forging a conceptual relationship between these two phenomena. First, we examine the definitions of nostalgia and heroism from scholars, laypeople, and across cultures, noting how the history of defining the two phenomena has shaped current conceptualizations. Second, we demonstrate how nostalgic experiences consist of reminiscences about our own personal heroism and about cultural role models and heroes. A review of heroism research, moreover, shows also that our recall of our heroes and of heroism is tinged with nostalgia. Third, we make linkages between heroism and nostalgia research focusing on functions, inspiration, sociality, and motivation. Nostalgia researchers have illuminated the functions of nostalgia implicating the self, existential concerns, goal pursuit, and sociality. Our review shows that heroism researchers invoke similar categories of hero functionality. Finally, we propose three areas of future research that can profit from the merging of nostalgia and heroism science, involving the mechanisms by which (a) heroism can fuel nostalgia, (b) nostalgia can promote heroic action, and (c) wisdom results from nostalgic reverie.

## Introduction

Each year over 100 countries around the world celebrate Mother’s Day. Commemorations of the day are replete with social media testimonials featuring people’s nostalgic remembrances of their mothers. These memorials underscore the heroic role of mothers in shaping our values, character, and behavior (Allison and Goethals, 2011). On Mother’s Day, stories abound of mothers passing on wisdom and fundamental truths about life to their children. Remembrances of mothers also include tales of our mom role-modeling the highest standards of human conduct, including exceptional acts of selflessness and sacrifice. Moreover, mothers are celebrated for motivating and inspiring us all to become our best selves. Mother’s Day memories also include accounts of mothers protecting and defending their children from danger. These four types of Mother’s Day nostalgic remembrances – wisdom, moral modeling, enhancing, and protection – correspond to the primary functions of heroism (Kinsella et al., 2015b; Allison, 2019). To our knowledge, there is no scholarship illuminating the relationship between the psychology of nostalgia and that of heroism. The purpose of this article is to propose some important theoretical connections between these two social psychological phenomena.

## Definitional Issues

At first blush, our examples of Mother’s Day testimonials point to obvious linkages between nostalgia and heroism. While the two phenomena share common ground, there are several striking differences. For example, nostalgia is defined as an emotion (Sedikides et al., 2015), whereas heroism is defined as a set of behaviors (Allison et al., 2017). Yet while nostalgia is an emotion, it implicates a surprisingly complex array of other psychological processes, including social behavior (Sedikides and Wildschut, 2019). Moreover, while heroism is a set of behaviors, it also encompasses a multitude of emotions and responses (Allison et al., 2019). We begin our analysis by comparing the definitions of nostalgia and heroism from the perspective of dictionaries, laypeople, scientific discovery, and diverse cultures.

### Dictionary Definitions

Dictionary definitions of nostalgia describe it as “a bittersweet longing for the past” (American Heritage Dictionary, 2020), a “pleasure and sadness that is caused by remembering something from the past and wishing that you could experience it again” (Merriam-Webster Dictionary, 2020), “a bittersweet yearning for the things of the past” (Wiktionary, 2020), and “a feeling of pleasure and also slight sadness when you think about things that happened in the past” (Cambridge Dictionary, 2020). These definitions emphasize sentimental longing or yearning, bittersweetness, and the positivity resulting from thinking about specific memories from one’s past. It is important to note that none of these definitions specify the exact nature of these circumstances. As it will become clear, we argue that much of the content of nostalgic recall centers on heroes and heroism.

Dictionary definitions of nostalgia, focusing on people’s recall of “things of the past,” may be alluding to ruminations about past positive events that could include heroic actions. Dictionary definitions of heroism, however, contain no allusions at all to nostalgic activity. Dictionaries describe heroism as “impressive and courageous conduct or behavior” (American Heritage Dictionary, 2020), “conduct especially as exhibited in fulfilling a high purpose or attaining a noble end” (Merriam-Webster Dictionary, 2020), “the display of qualities such as courage, bravery, fortitude, unselfishness” (Wiktionary, 2020), or “behavior directed toward achieving something very brave or having achieved something great” (Cambridge Dictionary, 2020). None of these definitions hints at the possible use of heroism as content for nostalgic remembrances, but they do refer to exceptional positive actions that may well be worthy of future nostalgic ruminations.

### Layperson Definitions

Hepper et al. (2012) asked participants to generate attributes of nostalgia and used a prototype methodology to identify 18 central features and 17 peripheral features. Central nostalgia features included the descriptors of *fond* and *rose-colored*. They also contained verbs such as *remembering*, *reminiscing*, *thinking*, and *reliving*. Moreover, central features included personally significant memories of childhood and social relationships. Central features also consisted of more positive feelings than negative ones, although they did contain a sense of longing, missing, and wanting to return to the past. Central features included nostalgia triggers such as keepsakes and sensory cues. Peripheral features, on the other hand, entailed such features as warmth, daydreaming, change, calm, regret, prestige, and lethargy. Overall, Hepper et al.’s results indicate that laypersons view nostalgia as a mostly positive, social, and past-oriented emotion. Their research suggests that lay conceptions of nostalgia correspond well with more formal dictionary definitions.

Nostalgic reverie typically has a redemptive component, moving from negative life circumstances to successful ones, and overcoming life circumstances (67 and 76% of the time in two studies; Wildschut et al., 2006). The self almost always plays a central role (87% of the time across two studies; Wildschut et al., 2006). Momentous events (e.g., finishing a marathon and graduating) also are common nostalgic recollections (34% of individuals in one sample; Wildschut et al., 2006). Memories of people are another common topic. Negative events tend to trigger nostalgic remembrances (Wildschut et al., 2006), suggesting that nostalgia can be used as a self-regulatory mechanism (Routledge, 2015).

Allison and Goethals (2011) asked participants to list traits of heroes and subjected these traits to factor and cluster analyses. The resultant categories revealed the “great eight” traits of heroes: *intelligent*, *strong*, *reliable*, *resilient*, *caring*, *charismatic*, *selfless*, and *inspiring*. Using a prototype analytic approach, Kinsella et al. (2015a) identified 13 central characteristics of heroes and 13 peripheral characteristics. Kinsella et al.’s central characteristics are *brave*, *moral integrity*, *conviction*, *courageous*, *self-sacrifice*, *protecting*, *honest*, *selfless*, *determined*, *saves others*, *inspiring*, and *helpful*. The peripheral characteristics of heroes are *proactive*, *humble*, *strong*, *risk-taker*, *fearless*, *caring*, *powerful*, *compassionate*, *leadership skills*, *exceptional*, *intelligent*, *talented*, and *personable*. These lay-definitions of heroism are consistent with those of the dictionaries we consulted. Moreover, although none of these heroic traits offers hints about their nostalgic qualities, the extremely high positive valence of these traits suggests exceptional attributes and actions that are life-changing and highly memorable.

Many of these heroic traits are moral or social traits (e.g., personable, caring, compassionate, and leadership), a pattern that is consistent with the social nature of most nostalgic reflections (Wildschut et al., 2006). Other heroic traits are also associated with momentous personal events that require effort, perseverance, and even bravery (e.g., courageous, powerful, exceptional, talented, and self-sacrifice). By definition, heroic behaviors should leave an indelible mark on the recipients of the behavior who may later be motivated to wax nostalgic about them. As with nostalgia, negative events tend to set the stage for heroic behavior, with a personal or societal crisis triggering the need for a hero and similar crises being shown to engender nostalgic ruminations (Sedikides and Wildschut, 2020).

### Scientific Definitions

Scholars over the years have shown a dramatic shift in their definitions of both nostalgia and heroism. For centuries, nostalgia was viewed as an indication of pathology, beginning with Hofer (1688/1934), who coined the term in his dissertation and conceptualized it as a neurological disease. Throughout most of the twentieth century, nostalgia was still viewed as a psychiatric or psychosomatic disorder (McCann, 1941). Nostalgic people were judged as depressed, showing “a regressive manifestation closely related to the issue of loss, grief, incomplete mourning, and finally, depression” (Castelnuovo-Tedesco, 1980, p. 110). Psychoanalysts concurred on “the importance of the preoedipal mother in the emotional developments of nostalgics” (Kleiner, 1977, p. 17), with nostalgia being regarded as “an acute yearning for a union with the preoedipal mother, a saddening farewell to childhood, a defense against mourning, or a longing for past forever lost” (Kaplan, 1987, p. 466). In short, scholars a century ago believed that nostalgia reflected an overattachment to one’s mother, a weakness that condemned the “sufferer” of nostalgia to a weakened, submissive, infantile, stereotypically “feminine” state.

By the turn of the millennium, researchers had parsed the positive side of nostalgia from its negative side (Hepper et al., 2012), paving the way for nostalgia’s current conceptualization as a functionally healthy emotion with far more positive effects than negative (Sedikides et al., 2006a,b). Still, we suspect that early conceptualizations of nostalgia as reflecting weakness and “femininity” helped to keep the focus on nostalgia’s emotional qualities rather than on its cognitive, motivational, and behavioral elements. Here, we see a significant departure from early scholarly treatments of heroism, which emphasized agency and power. If early conceptions of nostalgia were characterized by scholars as signaling weakness, femininity, and an overattachment to mothers, conceptions of heroism were characterized by the opposite tendency, namely, a bias toward strength and hyper-masculinization.

Heroism also has a storied history and has undergone a significant makeover in the eyes of scholars. Early conceptions of heroes emphasized the qualities of power, apotheosis, and masculinity (Hughes-Hallett, 2004). Heroism in antiquity was reserved exclusively for men who were venerated for their strength, courage, resourcefulness, and ability to slay enemies (Schein, 1984). The human tendency to assign god-like characteristics to heroic leaders can be traced to Beowulf and Achilles, and it later became manifest as the “divine right of kings” during the Middle Ages and Renaissance. This kind of thinking gave rise to the blatantly sexist *great man theory* of heroic leadership (James, 1880). The progenitor of this masculinization of leadership, Carlyle (1841) believed that “worship of a hero is transcendent admiration of a Great Man” (p. 19). From his perspective, all human beings “in some sense or other, worship heroes; that all of us reverence and must ever reverence Great Men” (p. 24). The allure of heroism taps into a deeply rooted archetype of god-like individuals who are “the creators” and “the soul of the whole world’s history” (p. 6). Hero worship, from Carlyle’s perspective, “is the deepest root of all; the tap-root, from which in a great degree all the rest were nourished and grown…. Worship of a hero is transcendent admiration of a Great Man” (p. 18–19). Carlyle wrote that heroes possess “a sort of savage sincerity ‐‐ not cruel, far from that; but wild, wrestling naked with the truth of things” (p. 193). As a result, “the history of the world is but the biography of great men” (p. 12). We make what should be the obvious observation that Carlyle and many other early leadership scholars refer to great “men,” never “persons.”

Freud (1922) also contributed his views on heroism, again with a strong masculine bias. Freud argued that the prototypical heroic leader of early human groups, “at the very beginning of the history of mankind, was the *Superman* whom Nietzsche only expected from the future…. The leader himself need love no one else, he may be of a masterly nature, absolutely narcissistic, but self-confident and independent” (p. 3). These “primal horde leaders,” observed Freud, become deified in death. Because we respond to charismatic leaders with reverence and awe, leaders who invoke religious feelings and ideation are viewed as especially charismatic. Freud believed that human beings gravitate to groups and crave heroic leadership that is powerful, always male, and charismatic. Invoking an evolutionary basis for male heroic leadership, Freud argued “that the primitive form of human society was that of a horde ruled over despotically by a powerful male” (p. 122).

If scholars were guilty of a male bias, it may have stemmed in part from the reality that throughout most of human history, heroism has been an activity reserved for men and denied to most women. Men’s advantage in the heroic realm has likely stemmed from their greater physical prowess, highly entrenched patriarchal social forces, and the restrictions that women’s reproductive duties often place on their activities (Wood and Eagly, 2002; Becker and Eagly, 2004). Most classical descriptions of heroism have thus emphasized male behavior and masculine attributes and, until recently, most theories of heroism have been gender-biased toward the male perspective. Thus, we argue that conceptually merging the two research areas of nostalgia and heroism may help each one avoid the pitfalls of being either too stereotypically masculine or feminine.

Heroism is defined by most contemporary researchers as extreme prosocial behavior that is performed voluntarily, involves significant risk, requires sacrifice, and is done without anticipation of person gain (Franco et al., 2011; Allison et al., 2017). Not only is heroic action inclusive with regard to gender but also expanded definitions of heroism that include more stereotypically feminine and communal traits suggest that women can be more heroic than men (Hoyt et al., 2020). While most heroism scholars favor efforts to develop an objective definition of heroism, other scientists have pushed back against extreme objectivity, arguing that heroism is ultimately a mental and social construction and therefore in the eye of the beholder (Allison and Goethals, 2011). What’s important is the label that people assign to the hero – their perception of heroism – more than a strict cataloguing of that person’s actions as heroic. The subjectivity of heroism is an important issue in considering whether people nostalgize about heroes. Older Americans, for examples, can share a longing for the wisdom of Martin Luther King, Jr., but on Mother’s Day one person’s mother differs from that of another person. Thus, we are more likely to agree about the identity of specific cultural heroes than about our personal heroes. For this reason, the distinction between culturally shared nostalgia and personal nostalgia is an important one, especially when considering the heroic content of nostalgia.

### Cross-Cultural Definitions

Hepper et al. (2014) measures conceptions of nostalgia in a range of cultures spanning 18 countries (e.g., Australia, Chile, China, Ethiopia, Germany, India, Japan, Uganda, Romania, and United States) across five continents. Nostalgia is universally regarded as an emotion, especially one of longing. It entails remembering or reminiscing about fond memories from the past. These memories have personal relevance or involve relationships with others. There was also considerable cross-cultural agreement regarding the interrelations among the 35 features. A factor analysis of the pooled correlation matrix revealed three factors. The primary factor, longing for the past, comprises cognitive, motivational, and contextual features of nostalgia along with longing and loss. The second factor, negative affect, consists of peripheral negative affective features. The third factor, positive affect, contains central and peripheral affective features – both general (e.g., emotion and relationships) and positive (e.g., warmth and happiness) ones.

Whereas nostalgia is considered as a universally shared emotional experience, heroism appears to be more culturally specific. Cross-cultural studies of heroism are rare. One study, conducted by Spyrou and Allison (2019), compared American, Greek, and Indian participants’ conceptions of heroes and hero attributes. The results showed some overlap in hero categories (e.g., social activists), but Indians tended to list celebrities and actors as heroes to a far greater degree than did Greeks and Americans. Greeks, moreover, were more likely to list war heroes and heroes from antiquity than the other two nations. Americans were more likely to list athletes, superheroes, and scientists. Differences in heroic attributes showed that Indians valued agency, piety, and charisma, whereas Greeks and Americans tended to value the heroic traits of altruism and integrity along with agency. The greater subjectivity and cultural varieties of heroism compared to nostalgia may explain why there are so few cross-cultural studies of heroism. Nostalgia may implicate universal psychological processes involving emotional expression, whereas the content of heroism may be more person-specific and culture-specific.

## Heroism in Nostalgic Content

In their seminal article that opened the floodgates for nostalgia research, Wildschut et al. (2006) found that “the two most common objects of nostalgia were persons and momentous events” (p. 980). This finding suggests that nostalgic remembrances have heroic content. It seems reasonable that many of the “persons” about whom we nostaligize are people we most admire and place on a heroic pedestal. Wildschut et al. found that the two most prevalent content items across two studies were close others (friends, family, and romantic partners) and momentous events. These investigators did not specify the exact nature of those close others, unfortunately. But they did discover higher nostalgia ratings for family among participants with negative affect and loneliness. In short, negative emotions tend to trigger nostalgia and, in particular, people become more nostalgic for close family members during difficult emotional times. Additional research by Routledge (2015) has shown that a large proportion of nostalgic content includes memories of activities involving close nuclear family members. This finding, coupled with the discovery of Allison and Goethals (2011) that over 40% of their survey respondents listed either their mothers or fathers as their heroes, suggests that our heroes tend to occupy a significant portion of nostalgic content.

Wildschut et al. (2006) also discovered that “descriptions of nostalgic experiences typically featured the self as a protagonist in interactions with close others (e.g., friends) or in momentous events” (p. 975). The perception of the self as a hero has not been studied by heroism scientists, possibly because strong social norms exist that discourages pronouncements of one’s own heroism (Worthington and Allison, 2018). Still, private ruminations and daydreams of one’s own past heroic accomplishments, even embellished ones, are likely to be common (Greenwald, 1980). Wildschut et al. found that most nostalgic content about the self was redemptive in nature, with redemption defined as occurring when “the narrative progresses from a negative life scene to a positive or triumphant one” (p. 976). Heroism scientists have found that redemption is one of the central characteristics of a heroic life (Allison and Goethals, 2011). It therefore seems reasonable that much of nostalgia features one’s past self-occupying the role of a hero. McAdams (2014) has reviewed many studies highlighting the powerful role of self-redemption in the crafting of one’s personal identity. McAdams finds that people are strongly motivated to transform their suffering into a positive emotional state, moving from pain to redemption. Self-redemption is also the centerpiece of the classic hero’s journey in storytelling (Allison et al., 2019).

We believe that a promising area for future research resides in exploring whether people nostalgize about heroes. This research should tap directly into nostalgic memories and should ask people to rate the degree to which a person or persons in their memories are heroic. If nostalgic remembrances an infused with heroic elements, then this research should show that the people about whom we are nostalgic tend be viewed as heroic. Moreover, we envision future researchers conducting an experiment that directly manipulates the target of a nostalgic remembrance, with some participants recalling people who have been important to them (“others” as heroes condition) and other participants recalling “a past event when you accomplished something you are proud of” (“self” as hero condition). We would expect people to rate the persons in their nostalgic memories as heroic. In addition, we might predict that nostalgizing about others as heroes would produce different psychological consequences compared to nostalgizing about the self as a hero. Because past investigations of nostalgia (e.g., Wildschut et al., 2006; Sedikides et al., 2015) have shown that nostalgia reduces loneliness, we would expect that this loneliness effect might only apply to nostalgia about others as heroes compared to nostalgia about the self as a hero. Moreover, because past nostalgia research has also shown that nostalgia can make people more goal-oriented (Sedikides et al., 2018; Sedikides and Wildschut, 2020), we would expect that this goal-orientation effect would be stronger when people nostalgize about the self as a hero than when nostalgizing about others as heroes. Future studies such as these would suggest that nostalgia about others as heroes procures different psychological benefits than nostalgia about the self as a hero. Establishing an empirical link between nostalgia and heroism may offer exciting scientific extensions of both scholarly areas.

## Nostalgia and Heroism: Motivation, Inspiration, and Prosociality

Since seminal article of Wildschut et al. (2006), nostalgia has been shown to fulfill several significant psychological functions. Sedikides and Wildschut (2019), for example, have demonstrated that nostalgic experience tends to trigger both inspiration and motivation in ways that appear to parallel the manner in which heroism inspires and motivates (Allison and Goethals, 2016). Nostalgia may therefore play a role in energizing and directing heroic feelings and intentions, thereby increasing the likelihood of risky and unusual actions associated with heroism. We illuminate the connection between these benefits of nostalgia and heroism below.

### Motivation

In their review of the motivational benefits of nostalgia, Sedikides and Wildschut (2020) distinguish among three kinds of motivational benefits: *generalized*, *localized*, and *action-oriented* motivation-based benefits. Generalized motivational benefits include the finding that nostalgia increases one’s sense of youthfulness, with people experiencing lower subjective age, more alertness, and increased energy. Allison and Goethals (2014) proposed an “energizing” function of heroism, and there are self-report data supporting this assertion (Allison and Goethals, 2011; Kinsella et al., 2015b). Sedikides and Wildschut found that nostalgia also promotes inspiration, engendering a sense of new possibilities, and it encourages financial risk-taking. In heroism science, Kohen et al. (2017) review several case studies of heroic risk-taking, concluding that heroes are role-models for prosocial risk-taking behavior. Franco et al. (2011) noted that the risk-taking aspect of heroism is what makes heroism especially desirable and emotionally moving. Allison and Goethals (2016), moreover, have argued that heroes who help in emergency situations provide people with mental scripts for performing similar heroic acts in their own lives.

The second type of motivational benefit of nostalgia identified by Sedikides and Wildschut (2020), localized motivation, includes a boost in people’s growth orientation, including an increase in growth-oriented self-perceptions and behavioral intentions. Nostalgia enhances intrinsic motivation, and strengthens one’s desire to pursue important goals. A number of heroism scholars have uncovered the similar tendency of heroism to invoke idealized versions of the self, to recover from past personal wounds, and to develop and pursue meaningful personal goals (e.g., Efthimiou et al., 2018; Williams, 2018; Bray and James, 2019; Ross, 2019). One could argue that exposure to heroism provides inspiration for growth, and that nostalgic ruminations about such heroism offers reminders about our growth-oriented goals and opportunities.

The third category of motivational benefit of nostalgia identified by Sedikides and Wildschut (2020), action-oriented motivation, stems from the finding that nostalgia galvanizes people’s desire to retain their memberships in organizations, increases people’s willingness to help others, and reduces people’s willingness to engage in self-destructive behaviors such as gambling and smoking. In the science of altruism and heroism, a number of investigators have found that the act of witnessing someone helping another person increases people’s willingness to help others (Latane and Darley, 1968; Fischer et al., 2011). In addition, Keck et al. (2018) have shown how exposure to heroic actions promotes post-traumatic growth, such that individuals inspired by heroes tend to adopt healthy new beliefs and values, view themselves and the world in a more positive manner, acquire wisdom, and experience a greater appreciation for life. In effect, witnessing acts of heroism helps to transform survivors of trauma into the heroes of their own life journeys.

We argue that nostalgia can play a key role in promoting action-oriented motivations aimed at heroic self-recovery and growth. Nostalgia instigates approach-motivation, “mixing memory and desire,” in the words of Eliot (1888). Specifically, nostalgic memories of one’s best self tend to motivate people to pursue a more idealized self in the future. Sedikides and Wildschut (2020) also found that nostalgia also boosted self-esteem. Such an augmentation of the self could reasonably contribute to people acting in heroic ways in the future. Nostalgia promotes a growth orientation (e.g., state authenticity or intrinsic self-expression, growth-oriented self-perceptions, and behavioral intentions), galvanizes intrinsic motivation, and strengthens the pursuit of one’s important goals. This action-oriented motivation cements an employee’s resolve to stay with the organization (i.e., weakens turnover intentions), increases the propensity to help and actual helping, and contributes to behavior change. In short, nostalgia and heroism may work in tandem to promote a better self and a better society.

The nostalgia-heroism link is nicely illustrated in a study conducted by Abeyta et al. (2014), who gave American undergraduates either a nostalgic-event or an ordinary-event writing prompt. Three coders rated the ensuing narratives on three categories. The first category included social content (i.e., social interactions and relationships), the second included more specific attachment-related content (i.e., feeling loved, protected, and trusted by others), and the third included agency (i.e., personal competence, success, and power). The coders also rated the presence of positive and negative feelings. Abeyta et al. found that nostalgic (compared to ordinary) narratives contained more references to all three categories, attesting to the relevance of sociality and identity for the nostalgic experience. The nostalgic (compared to ordinary) narratives were characterized by more positive than negative feelings, and more feelings in general. These findings are consistent with hero research showing that heroism implicates these same prosocial categories. A heroic act is a social activity promoting the galvanization of relationships (Allison, 2019), an attachment-oriented activity involving the protection of loved ones (Kinsella et al., 2015b), an activity of potency and agency (Hoyt et al., 2020), and an activity implicating feelings of warmth, nurturance, and care (Kinsella et al., 2017).

### Inspiration and Prosociality

Nostalgia may be one source of inspiration on the journey to heroic behavior. Stephan et al. (2015) found that individuals who experience nostalgia more often (i.e., nostalgia proneness) report feeling inspired more frequently and more intensely. Moreover, this association is causal: induced nostalgia – recalling a nostalgia memory relative to an ordinary (control) memory – heightens both general and specific inspiration for exploratory endeavors. Several additional studies that investigated the mechanism underlying this connection found a serial mediation effect, in which nostalgia increases feelings of social connectedness, which raise self-esteem, which increase inspiration. Does nostalgia-induced inspiration lead to any tangible motivation to pursue action? In one experiment by Stephan et al., individuals who engaged in nostalgic (vs. ordinary) reverie and wrote down their most important goal and their motivation to pursue that goal, nostalgia increased inspiration, which in turn increased intentions to pursue that most important goal. Currently, there is no heroism science research that points to the causal sequence of heroism engendering social connection, self-esteem, and inspiration. Given all the linkages between nostalgia and heroism that we have reviewed, it seems likely that our ideations about heroes play an important role in this psychological process.

In heroism science, there is research showing that heroic underdogs engender inspiration. When we encounter underdogs who enjoy unexpected success, we tend to identify with them, root for them, and judge them to be highly inspiring when they triumph (Kim et al., 2008; Allison and Burnette, 2009; Davis et al., 2011). Kinsella et al. (2015b) report data suggesting that the inspiring quality of heroes is what sets heroes apart from altruists, helpers, and leaders. When asked which one trait of heroes is the most important, people report that the trait of *inspiring* is the most telling attribute of a hero (Allison and Goethals, 2011). The finding that charisma is a central trait of heroes underscores the idea that heroes move us and inspire us (Kinsella et al., 2015a).

Reading or listening to tales of heroism has been shown to produce important psychological benefits (Allison et al., 2019). In classic hero mythology, the hero is separated from their safe, familiar world and thrown into dangerous, unfamiliar circumstances (Campbell, 1949). The hero is ill-equipped for the journey and is humbled to discover that they are missing important inner qualities such as self-confidence, courage, resilience, compassion, or wisdom. Encountering villains and setbacks, the hero receives help from allies and mentors who guide the hero toward personal transformation. Allison et al. (2019) describe six types of personal transformations that heroes undergo: mental, moral, emotional, spiritual, physical, and motivational. The metamorphoses of the hero foster developmental growth, promote healing, cultivate social unity, and advance society (Allison and Goethals, 2016). The popularity of novels, plays, and movies in which these heroic transformations occur can be traced to the inspirational benefits of the heroic growth that we witness in these stories (DuBois, 2019).

The final stage of the classic hero’s journey, involving the hero giving back what they have learned to society, underscores the idea that heroism is fundamentally prosocial rather than selfish. Several converging lines of research have demonstrated that nostalgia, at both the trait and state level, are associated with prosocial motivations and behaviors (Sedikides et al., 2015). For example, nostalgic individuals picked up more “accidentally” dropped pencils than individuals in a control state (Stephan et al., 2014). Nostalgic individuals gave more to charity than individuals in a control condition (Zhou et al., 2012) and individuals nostalgic for their university alma mater donated more frequently and in higher amounts (Green et al., 2020, under review) than those lower in university nostalgia. Nostalgia is associated with empathy (particularly affective, relative to cognitive, and empathy), which appears to mediate this link (Juhl et al., 2020), and attachment security mediates the link between trait nostalgia and increased empathy.

Nostalgia appears to inhibit some types of antisocial behaviors as well as activate prosocial behaviors. Nostalgic reminiscences are fundamentally social, warm, and close. As such, they may blur some boundaries between social groups, particularly when a reminiscence is shared with a wider, superordinate social group, which may help the nostalgizer assimilate elements of an outgroup. Two experiments (Turner et al., 2018) found that young people experiencing nostalgia were more likely than those in a control group to indicate a greater sense of overlap with older adults and greater social connectedness, which in turn fostered more positive attitudes (less prejudice) toward older adults. This reduction in prejudice elicited by nostalgia appears to be robust, having been extended to more positive views of immigrants (Gravani et al., 2018), and the mentally ill (Turner et al., 2018), among other groups. Thus, as heroic behavior may require both the inhibition of selfish and parochial attitudes and actions as well as the motivation and activation toward extraordinarily selfless behaviors, nostalgia may lubricate both aspects.

### Collective Nostalgia

Nostalgia exists at the collective level (Wildschut et al., 2014), conforming to the requirements of a collective or intergroup emotion, as articulated by intergroup emotions theory (Mackie et al., 2000). That is, collective nostalgia directs behavior toward the group, can be distinguished from personal nostalgia, and is correlated with the degree of identification with the group (Wildschut et al., 2014). Some heroic behavior may be activated by the *personal self* (i.e., unique motives and traits), but other *heroic behavior* may be activated by the collective self (i.e., identification of the self as part of a collective with shared traits, goals, and history). For example, Rosa Parks’ decision to refuse to comply with a request to change seat may have been influenced by her identification as an African American. To the extent that the collective self-motivates heroism, the analogous collective emotions, particularly collective nostalgia (e.g., reflecting on the unique cultural history of one’s group) may be critical determinants of this type of heroism.

National nostalgia comprises the vast majority of collective nostalgia research (Smeekes, 2015). While national nostalgia can elicit more positive feelings toward ingroups, it can also lead to negative attitudes about outgroups (e.g., anti-immigrant feelings; Smeekes et al., 2014), more parochial buying habits, etc. These links are not found for personal nostalgia, which typically correlates only modestly with national nostalgia. Smeekes (2015) has reviewed a considerable body of research pointing toward the potential destructive effects of national nostalgia, such as negative prejudicial attitudes behaviors directed toward citizens residing outside of one’s own national identity. History has taught us that while Adolf Hitler was a hero to the German people for boosting their national pride, his nostalgia for German greatness went to such an extreme that it led to genocidal attacks on outgroup members. Sedikides et al. (2008) induced collective nostalgia in Greek participants by having them write or read about Greek music and cultural traditions. Afterward, the participants indicated a preference for their Greek heritage, television shows, and consumer products. But this pro-Greek benefit of nostalgia had a negative consequence, as participants disparaged foods and products that were *not* Greek. The point we wish to make here is that while nostalgia and heroism can inspire people to become their best selves, it can also inspire people to become their darkest and worst selves.

## Heroism as Propulsion For Nostalgic Wisdom

Our review of the literature in the nostalgia and heroism fields shows that wisdom has received scant attention from nostalgia scholars, whereas the wisdom benefits of heroism has been the subject of considerable analysis. Consensus regarding the construct of wisdom is hard to come by, though most scholars agree that it involves harnessing and applying knowledge to effectively address real-world issues (Sternberg, 1998; Grossmann et al., 2010), typically for the common good (Webster, 2003; Sternberg, 2004), requiring perspective-taking and ego-decentering (Ardelt, 2003; Grossmann, 2017). Proposed facets of wisdom include emotion regulation and openness (Webster, 2003), reflectiveness (Ardelt, 2003), ego decentering (Grossmann, 2017), and many more. These scholarly approaches to wisdom coincide with dictionary definitions of wisdom as “the ability to judge what is true, right, and lasting” (American Heritage Dictionary, 2020) and “the ability to use knowledge and experience to make good judgments and decisions” (Cambridge Dictionary, 2020).

Heroism researchers have examined the ways in which heroes and hero narratives fulfill a number of important psychological and life-enhancing functions that relate to wisdom. In their analysis of the impact of hero stories and narratives throughout history, Allison and Goethals (2014) demonstrate how heroism offers wisdom to persons and groups (Cajete et al., 2010; Sternberg, 2011; McAdams, 2014). Stories crystalize abstract concepts and endow them with contextual meaning (Boje, 1995). Gardner (1995) and Sternberg (2011) point to numerous examples of heroic leaders using the persuasive impact of storytelling to win the minds and hearts of followers. Stories are not just tools of social influence directed toward others; they also can precipitate self-change. As mentioned earlier, McAdams (2014) has shown how personal self-narratives play a pivotal role in shaping our life trajectories and maintaining our subjective well-being. Stories offer vivid, emotionally laden capsule summaries of wisdom for which the human mind was designed (Wyer, 1995). Price (1978) has even asserted that “a need to tell and hear stories is essential to the species *Homo sapiens* – second in necessity apparently after nourishment and before love and shelter” (p. 3).

Allison and Goethals (2014) argued that hero narratives fulfill two principal psychological functions: an *epistemic* function and an *energizing* function. The epistemic function refers to the knowledge and wisdom that hero stories impart, whereas the energizing function refers to the ways that hero stories offer inspiration and promote personal growth. The epistemic benefits of heroism reside in heroic actions providing scripts for prosocial action, revealing fundamental truths about human existence, unpacking life paradoxes, and cultivating emotional intelligence. Stories of heroic action impart wisdom by supplying mental models, or scripts, for how one could, or should, lead one’s life. It seems reasonable that these heroic role models who supply such wisdom would be useful subjects of nostalgic reminiscences.

Hero stories reveal truths and life patterns that our limited minds have trouble understanding using our best logic or rational thought. Allison and Goethals (2014) have used the term *transrational* to describe these phenomena, in that these challenging truths tend to defy comprehension using conventional, logical methods. Transrational phenomena that commonly appear in hero stories include *suffering*, *love*, *paradox*, *mystery*, *God*, and *eternity*. These phenomena beg to be understood but tend to resist a full understanding using logic or reason. Hero storytelling has the ability to reveal the secrets of the transrational. These heroic tales help us to think transrationally in at least three ways: hero stories reveal deep truths, illuminate paradox, and develop emotional intelligence.

First, with regard to deep truths, we should note that the great mythologist Campbell (1949) devoted his entire career to championing the idea that hero myths reveal life’s deepest psychological truths. Truths are considered deep when their insights about human nature and motivation are not only profound and fundamental but also hidden and nonobvious. Campbell (1949) believed that most readers of mythic hero stories remain oblivious to their deep truths, their meaning, and their wisdom. Deep truths contained in hero myths are difficult to discern and appreciate because they are disguised within symbols and metaphors. As a result, readers of mythology underestimate the psychological value of the narratives, prompting Campbell to proclaim that “mythology is psychology misread as biography, history, and cosmology” (p. 256).

Hero stories convey deep truths by sending us into *deep time*, meaning that the stories have a timeless quality that connects us with the past, the present, and the future. Phrases such as, “Once upon a time,” “A long time ago in a galaxy far, far away,” and “they lived happily ever after,” are examples of storytelling devices that signal the presence of deep truths embedded in deep time. By grounding people in deep time, hero stories reinforce ageless truths about human existence. Classic hero stories and fairy tales tend to emphasize bygone times, saturating these narratives with nostalgic qualities. We suspect that these hero stories are designed to evoke nostalgia in order to underscore the wisdom contained in them.

It is easy to see how personal nostalgic reverie is connected to deep time. When we nostalgize, we are hurled back to a time when our deepest needs were most meaningfully satisfied, including, we would argue, the need to meet the challenges of a present situation by absorbing wisdom from past heroic role models. Past nostalgized heroes supplying us with wisdom need not be real people; they may be heroes from fictional stories about which we have fond memories. For example, the first author of this article recalls many times in his life when he has drawn wisdom and inspiration from the character of Huckleberry Finn, who demonstrated a courageous, adventurous spirit and an endearing innocence in his worldview. During those few times in his life when the author was able to experience a fleeting facsimile of Huckleberry Finn’s heroic life, this experience itself became the subject of the author’s future nostalgic reverie. We suggest that memories of our past self – our prideful accomplishments or hard-wrought redemptions – could be considered a source of heroic wisdom that we need to alleviate dark moods or to spur us into positive action.

In addition to deep truths and deep time, wisdom from hero stories also derives from *deep roles* in our human social fabric. Moxnes (2013) identified the deepest social roles in hero tales as archetypal family roles that include mother, father, child, maiden, and wise old man. Family role archetypes occupy pervasive character roles in classic hero mythology, where kings and queens, parents, stepparents, princesses, children, and stepchildren abound. Moxnes’ research shows that even if these deep role characters are not explicit in hero stories, human beings will project these roles onto the story characters. His conclusion is that the family unit is an ancient device for understanding our social world. From these considerations, we can understand how and why nostalgia content has been found to include many close family members (Wildschut et al., 2006; Routledge, 2015). We derive wisdom from family members occupying these deep social roles, and nostalgic remembrances offer us reminders of this wisdom. Batcho (2018) uncovered evidence for the transmission of this type of wisdom from close family members, analyzing the memoirs of Ukrainians who took part in resistance movements during the Second World War. She found that when her participants waxed nostalgic about their childhood values handed down to them from their parents, such nostalgia led many to risk their lives to engage in a dangerous freedom struggle.

Another form of heroism-based wisdom lies in the epistemic value of paradoxical truth. As author G. K. Chesterton once observed, *paradox is truth standing on its head to attract our attention*. Hero stories shed light on meaningful life paradoxes (Franco et al., 2011; Allison and Goethals, 2014). Throughout human history, the process of unpacking the value of paradoxical truths has been most effectively revealed through heroic storytelling (Rohr, 2011). Campbell (1949) argued that hero stories are saturated with paradoxical truths, including the idea that suffering can lead to enlightenment and that leaving home allows the hero to discover home. The first author of this article often nostalgizes about his grandmother, who passed down the paradoxical truth that one must give love away in order to receive it. The counterintuitive nature of paradoxical truths makes heroic storytelling, and our nostalgic remembrances of the heroic storyteller, a powerful source of wisdom.

The final type of wisdom that nostalgia may provide is the wisdom of emotional intelligence, defined as the ability to identify, understand, use, and manage emotions (Caruso et al., 2014). Bettelheim (1976) argued that children’s fairy tales are useful in helping people, especially children, understand emotional experience. With their many dark, foreboding symbols and themes, such as witches, abandonment, neglect, abuse, and death, these heroic fairy tales allow people to experience and resolve their fears. Bettelheim believed that even the darkest of fairy tales, such as those by the *Brothers Grimm*, help people to achieve clarity about confusing emotions. The hero of the story emerges as a role model by demonstrating how one’s fears can be overcome. A striking example can be found in the *Harry Potter* novels, which have been shown to help both children and adults face their anxieties, increase their empathy, and grow emotionally (Gibson, 2011; Stetka, 2014). In his classic book, *The Denial of Death*, which spurred considerable work on terror management theory, Becker (1973) argued that merely witnessing a heroic act, either in person or in literature, helps buffer anxiety and existential terror. In short, we may learn about the wise use of one’s emotions through heroic storytelling and through nostalgizing about those stories and the heroes in them.

## Conclusion: Three Future Research Directions

We began this article by noting the significance of Mother’s Day as an example of a cultural event honoring a hero who is also a likely target of frequent nostalgic reminiscences. If our mothers and other close family members can be a source of both heroism and nostalgia, then it would seem to be a fruitful exercise to identify and develop connections between these two psychological and behavioral phenomena. We noted that the definitions of the two phenomena suggest convergences focusing on the attributes of motivation, inspiration, and prosociality. The history of nostalgia and heroism, moreover, suggests a fascinating bias that may have kept the two research literatures apart for many decades. Nostalgia’s historical emphasis on over-emotionality, over-attachment, and weakness endowed it with a stereotypically feminine reputation, whereas heroism’s historical emphasis on agency, apotheosis, and leadership endowed it with a stereotypically masculine standing. Recent research on nostalgia that has discovered its potency (Sedikides and Wildschut, 2020), and heroism research that has unveiled the caring and nurturant side of heroic acts (Hoyt et al., 2020), have appropriately androgenized the two phenomena, thus smoothing the way for their theoretical integration.

We propose three areas of future research that may profit from the merging of nostalgia and heroism science. The first area ripe for further study focuses on identifying the mechanisms by which heroism can fuel nostalgia. The second promising area centers on the reverse causal direction, namely, the processes by which nostalgia might promote heroic action. Finally, we believe that future investigators may wish to explore the mechanisms underlying the acquisition and reinforcement of wisdom as yet another positive consequence of nostalgic experience.

### Heroism as Fuel for Nostalgia, Its Benefits, and Its Drawbacks

In the United States, the very existence of holidays throughout the year, such as Mother’s Day, Father’s Day, Veteran’s Day, and President’s Day, underscores the degree to which human societies create culturally rich infrastructures that engender nostalgia about our heroes. The longing for our past heroes is illustrated through heroic representations on our statues, our monetary currency, and named buildings, roads, schools, and cities. People need heroes and have a romantic longing for them (Goethals and Allison, 2019). The downside of this romanticization of heroes is that it leads to distorted thinking about the past, with selective attention given to desired aspects of past living over the drawbacks. Allison and Goethals (2020) have argued that Donald Trump’s “Make America Great Again” (MAGA) movement is an example of an immature narrative that uses heroism to divide people rather than unite them. Although the narrative is simplistic, it still paints a much-needed big cosmological picture for people, inside of which they can feel safe and secure. Trump has embraced the role of the heroic Messiah delivering the nostalgic message of reclaiming former greatness, calling himself “the chosen one.” That a significant portion of Trump’s supporters literally believe he was an answer to their prayers, and that he was divinely chosen to protect a Christian nation, speaks to the power of heroism-guided nostalgia (Whitehead et al., 2018). It also speaks to the unfortunate truth that there may be as many downsides to heroic nostalgia as there are benefits.

When we closely examine the MAGA movement, we can see how all four words of this slogan invoke a powerful cosmology, tinged with nostalgia, designed to move and to mobilize people. First, the word “Make” taps into the need for agency, the need to fashion something, to take control, to assume power, and to take action. This is very heroic imagery. The second word in MAGA, “America,” summons a powerful collective identity, the group to which people belong, the group that is loved and cherished and needs heroic protection at all costs. The third word, “Great,” conjures a sense of transcendence, of bigness, of superiority, along with a nostalgic longing for past national exceptionalism. Finally, the fourth word in MAGA, “Again,” implies the need to restore a sense of deep time, the need for America’s greatness to be eternal, though activating this national nostalgia can prompt derogation or prejudice against outgroups in addition to more positive views of ingroups (Smeekes, 2015; Sedikides and Wildschut, 2019). Thus, “Make America Great Again” is ingeniously crafted to stir people’s thirst for a heroic mythology that supplied esteem, bigness, and transcendence in the past and can continue to do so in the future.

If Donald Trump represents the misuse of heroism in fueling nostalgia, then we would be remiss if we failed to underscore the obvious constructive use of heroism in producing psychological benefits. As the initial investigation of Wildschut et al. (2006), close family members and friends comprised much of their participants’ nostalgic content. In this article, we have shown how remembering our heroes and reading about heroic actions can offer people protection benefits, enhance their well-being, provide moral modeling, supply mental scripts for positive action, and hand down timeless wisdom. Do heroes themselves confer these benefits or is it the act of nostalgizing about heroes that confers the benefits? The answer may be both, as present-day heroes, influencing us in the here and now, may spur positive emotions and actions. Nostalgic reminiscences about past heroes, in turn, may produce the same benefits, perhaps even to a greater degree if motivations to view history in a particular way are strong. Future research may profit from untangling these possibilities.

We propose that there may be a universal link between the desire to be a hero and one’s cultural values, and we further suggest that nostalgia plays a central role in establishing this link (Shahar, 2013; Israeli et al., 2018). Shahar (2013) identified three types of heroic self-representations: self-as-savior, self-as-conqueror, and heroic-identification. These self-images can be psychologically healthy ones if a person enjoys healthy early-life attachments and self-worth. But if early life experiences compromise healthy attachments or self-worth, the self-as-savior identity can emerge that manifests as an over-responsibility to help others. Moreover, a sense of “compulsive heroism” (Yalom, 1980) can emerge in the form of self-as-conqueror, marked by a need to overcome any and all challenges in the service of others. The third heroic self-representation, heroic identification, emerges from the role of societal norms in promoting the myth of heroism that encourages individuals to embrace heroic idols (Campbell, 1949). This third representation is also prone to pathology.

According to Shahar (2013), individuals with all three of these self-identities are likely to draw from cultural heroes as guides for fulfilling their heroic aspirations. Israeli et al. (2018) invoke the Jewish-Israeli myth of the *Tzabar* as an example. The Tzabar is a culturally influential mythical symbol of heroism, representing the heroic qualities of courage, sacrifice, inspiration, victory, solidarity, and camaraderie. On the downside, it is also tinged with sexism, ethnocentrism, militarism, nationalism, and narcissism. Israeli et al. found that heroic self-representations are likely to draw from the Tzabar, the good and the bad elements, particularly when the holders of these self-representations are under duress. Shahar (2013) argued that individuals harboring these three heroic self-representations may be prone to psychopathology under extreme stress, especially those with heroic identification as their self-representation. These latter individuals may deny their stress, viewing any sign of weakness as “unheroic” and inconsistent with the heroic script found in hero mythology. This conceptualization suggests that nostalgia about the wrong kinds of heroes and heroic ideals can heighten psychopathological tendencies.

To test these ideas, Israeli et al. (2018) studied adults during their prolonged exposure to the 2014 Gaza War, “Operation Protective Edge,” which occurred in Israel between July 8, 2014 and August 26, 2014. Israeli citizens’ emotional states were measured while they endured extensive air strikes, ground fighting in Gaza, and continuous large-scale rocket fire from Gaza to Israel. The results showed that participants’ heroic identification affected them emotionally and behaviorally, and sometimes not in healthy ways. While Israeli heroic identification with past heroes has been shown to promote heroic behavior (Brook, 2016), it can also produce significant psychological maladjustment. When confronting the severe psychological challenges of war, people may identify with the ideal heroic image of the person who can conquer any difficult obstacle or who can endure any amount of stress to act heroically. Doing so can lead to actions that save the lives of one’s ingroup members, but the consequences of taking on the role of a hero can be significant increases in perceived stress, self-criticism, general psychopathology, maternal overprotection, dissociative depersonalization and absorption, PTSD severity, and attachment anxiety. Thus, the nostalgia-heroism link is likely a reflection of the cultural basis of heroism. Israeli et al. (2018) demonstrated, primarily through the Israeli culture, that cultural values may feed hero-based nostalgia.

### Nostalgia as a Mechanism for Promoting Heroism (and Villainy)

Over the past several years, nostalgia researchers have illuminated many behavioral benefits nostalgic feelings, with some of these benefits reflecting precursors to heroism or possible heroic actions themselves. Sedikides and Wildschut (2020), for example, found that nostalgia enhances people’s growth-oriented self-perceptions and behavioral intentions. Nostalgia bolsters our desire to pursue self-improvement goals, galvanizes our group loyalty, raises our willingness to help others, reduces our engagement in self-destructive behavior, motivates us to pursue a more idealized self, and boosts self-esteem and self-efficacy. Stephan et al. (2015) reported that individuals high in nostalgia proneness feel inspired more intensely and more often. Moreover, these concomitant benefits of nostalgia are causal, with induced nostalgia heightening both general and specific inspiration for risk-taking, exploratory endeavors. These studies have shown that nostalgia increases feelings of social connectedness, which in turn boosts self-esteem and inspiration. Nostalgia-induced inspiration, moreover, leads to heightened inspiration to pursue important self-goals, which in turn bolsters intentions to pursue those goals. All of these mechanisms of self-empowerment and collective engagement are likely to contribute to people acting in heroic ways.

We propose that nostalgia helps propel people on their personal heroic journeys. Whether intentional or not, Sedikides and Wildschut (2020) use the language of the mythic hero’s journey to describe the positive behavioral impact of nostalgia. “Nostalgia,” they wrote, “cultivates a planful and future-oriented mindset. It motivates and helps the individual to shape, in part, their destiny” (p. 78). Heroic protagonists in books and movies will often use their nostalgic memories of past personal heroes to propel them into heroic action. A prominent example is Forrest Gump, the eponymous hero who reflects back on his mother’s wisdom during times in his life when he seeks direction or needs an inspirational boost. Another powerful example, in the original *Star Wars* film, occurs during the movie’s climax when Luke Skywalker invokes the past wisdom of his hero, Obi-Wan Kenobi (“Use the force, Luke”), to extricate himself from a challenging situation. Nostalgia, we now know, is much more than mere emotion. It is grist for the heroic mill.

### The Acquisition of Wisdom as a Central Benefit of Nostalgia

We noted earlier that hero stories are packed with transrational phenomena that are best understood in the context of hero storytelling. We argue that heroic tales help us to unravel the mysteries of transrational life events by revealing deep truths, deep time, deep roles, the nature of paradox, and examples of emotional intelligence. Delving into scholarship on the nature of wisdom, we briefly review two measures of wisdom whose scale items reflect the wisdom gleaned from nostalgia, heroism, and these transrational phenomena.

One such measure of wisdom is *The Self-Assessed Wisdom Scale* (Webster, 2003) containing items that reflect four categories: nostalgic recollections, a reliance on the wisdom of others as heroes, the learning experiences of the self as a hero, and the development of emotional intelligence. The scale measures wisdom gleaned from nostalgia by including items such as, “I reminisce quite frequently,” “I often think about my personal past,” and “Recalling my earlier days helps me gain insight into important life matters.” The scale taps into wisdom gleaned from others with items such as “I’ve learned valuable life lessons from others,” and “I like to read books which challenge me to think differently about issues.” Wisdom gleaned from one’s own heroic journey is found in items such as, “I have overcome many painful events in my life,” and “Reliving past accomplishments in memory increases my confidence for today.” Emotional intelligence is measured by such items as, “I am tuned into my emotions,” and “I can regulate my emotions when the situation calls for it.” In short, the assessment of wisdom from this scale involves mechanisms implicated in the processing of hero narratives involving the interplay of nostalgia, others as heroes, the self as a hero, and emotional intelligence. We believe that it may be fruitful for future researchers to use this scale to investigate the ways in which nostalgia may promote the acquisition of hero-derived wisdom and emotional wisdom.

Another measure of wisdom, *The Three-Dimensional Wisdom Scale* (Ardelt, 2003), operationalizes wisdom along the three dimensions of cognition, reflection, and affect. The cognitive dimension taps into tolerance for ambiguity (e.g., “There is only one right way to do anything”), dogmatism (e.g., “In this complicated world of ours, the only way we can know what’s going on is to rely on leaders or experts who can be trusted”), need for cognition (e.g., “I try to anticipate and avoid situations where there is a likely chance I will have to think in depth about something”), and attitudes about reality (e.g., “It is better not to know too much about things that cannot be changed”). The reflective dimension contains items measuring perspective-taking (e.g., I try to look at everybody’s side of a disagreement before I make a decision”), empathy (“Before criticizing somebody, I try to imagine how I would feel if I were in their place”), and resentment (e.g., “When I look back on what has happened to me, I cannot help feeling resentful”). The affective dimension of the scale includes items measuring compassion (“It’s not really my problem if others are in trouble and need help”), acceptance (“I’m easily irritated by people who argue with me”), and helping (“If I see people in need, I try to help them one way or another”). These latter items, from both the reflective and affective components of the scale, would seem to represent measures of emotional intelligence and prosociality.

There is empirical evidence that the *Self-Assessed Wisdom Scale* (Webster, 2003) and the *Three-Dimensional Wisdom Scale* (Ardelt, 2003) are correlated with, and predict, wise behavior. Using both scales, Taylor et al. (2011) found that these two measures of wisdom predicted psychological well-being and the pro-relationship (and psychologically healthy) behavior of forgiveness. Participants’ wisdom scores predicted their environmental mastery (e.g., “In general, I feel in charge of the situation in which I live”), personal growth (e.g., “I am not interested in activities that will expand my horizons”), self-acceptance (e.g., “I like most aspects of my personality”), autonomy (e.g., “I tend to be influenced by people with strong emotions”), purpose in life (e.g., “I live one day at a time and do not really think about the future”), positive relations with others (e.g., “I feel like I get a lot out of my friendships”), and making peace with others (“I make peace with people who have hurt me”). Whether nostalgic remembrances can engender wisdom as measured by these scales remains an empirical question.

We conclude this article by revisiting one of the most storied heroes in Western literature, Odysseus, a man whose accomplishment and fame were built on the fusion of both nostalgia and heroism. Odysseus, as vividly revealed in Homer’s *Odyssey*, was a prototypical hero on the original hero’s journey. A wise leader of Ithaca, and reluctant to leave his family to fight in the Trojan War, he nevertheless exhibited courage and bravery throughout that war. However, he was best known as a cunning strategist. The Greeks would not have won the Trojan War without his recruiting Achilles and especially without his Trojan horse idea, which ultimately won the war. In addition to these agentic qualities, he exhibited more communal heroic traits on the long journey home. He protected his men as best he could from the Cyclopes Polyphemus and the sorceress Circe, he shrewdly navigated between Scylla and Charybdis, and he cleverly managed to listen to the Sirens’ song and live to tell the tale. His devotion to his wife and son kept him from giving up after a decade of tribulation. In short, Odysseus exemplified most of the traits of heroism identified by Allison and Goethals (2011) and Kinsella et al. (2015a).

Odysseus also was the original nostalgizer (Hepper et al., 2012; van Tilburg et al., 2018). It’s hard to imagine anyone suffering more (algos), striving for a decade to return to his home (nostos). His memories of Ithaca and his loved ones sustained him, inspired him, and motivated him to overcome immense challenges, temptations, and tragedies. He even turned down Calypso’s offer of immortality so that he could return to his family. We argue that it is no coincidence that nostalgia and heroism are intertwined in Odysseus. It was Odysseus’ nostalgia that catalyzed and motivated his extraordinary heroism during his decade-long journey home. We also emphasize that the journey of Odysseus laid the groundwork for future iconic heroes who longed to go home, from Dorothy in *The Wizard of Oz* to Simba in *The Lion King*. Nostalgia strikes at the heart of heroism. We hope that our analysis and integration of these two phenomena prove useful to future investigators.

## Data Availability Statement

The original contributions presented in the study are included in the article/supplementary material, further inquiries can be directed to the corresponding author.

## Author Contributions

All authors listed have made a substantial, direct and intellectual contribution to the work, and approved it for publication.

### Conflict of Interest

The authors declare that the research was conducted in the absence of any commercial or financial relationships that could be construed as a potential conflict of interest.
